# Terrestrial isopod community as indicator of succession in a peat bog

**DOI:** 10.3897/zookeys.176.2379

**Published:** 2012-03-20

**Authors:** Ivan Antonović, Andreja Brigić, Zorana Sedlar, Jana Bedek, Renata Šoštarić

**Affiliations:** 1 Faculty of Science, University of Zagreb, 10000 Zagreb, Croatia; 2 Department of Zoology, Faculty of Science, University of Zagreb, Rooseveltov trg 6, 10000 Zagreb, Croatia; 3 Department of Botany, Faculty of Science, University of Zagreb, Marulićev trg 20/II, 10000 Zagreb, Croatia; 4 Croatian Biospeleological Society, Demetrova 1, 10000 Zagreb, Croatia

**Keywords:** Edge, seasonal dynamics, pitfall trapping, predation, *Trachelipus rathkii*, *Protracheoniscus politus*, *Hyloniscus adonis*, tyrphoxenous species

## Abstract

Terrestrial isopods were studied in the Dubravica peat bog and surrounding forest in the northwestern Croatia. Sampling was conducted using pitfall traps over a two year period. Studied peat bog has a history of drastically decrease in area during the last five decades mainly due to the process of natural succession and changes in the water level. A total of 389 isopod individuals belonging to 8 species were captured. Species richness did not significantly differ between bog, edge and surrounding forest. High species richness at the bog is most likely the result of progressive vegetation succession, small size of the bog and interspecific relationships, such as predation. With spreading of *Molinia* grass on the peat bog, upper layers of *Sphagnum* mosses become less humid and probably more suitable for forest species that slowly colonise bog area. The highest diversity was found at the edge mainly due to the edge effect and seasonal immigration, but also possibly due to high abundance and predator pressure of the *Myrmica* ants and lycosid spiders at the bog site. The most abundant species were *Trachelipus rathkii* and *Protracheoniscus politus*, in the bog area and in the forest, respectively. Bog specific species were not recorded and the majority of the species collected belong to the group of tyrphoneutral species. However, *Hyloniscus adonis* could be considered as a tyrphoxenous species regarding its habitat preferences. Most of collected isopod species are widespread eurytopic species that usually inhabit various habitats and therefore indicate negative successive changes or degradation processes in the peat bog.

## Introduction

Peat bogs are a type of wetlands characterized by a high water table, low levels of nutrients, low pH values, and are dominated by *Sphagnum* mosses (Spitzer and Danks 2006). They are widely distributed in specific climatic regions where climates are cool and precipitation is relatively high, mostly in the boreal zones of Europe, Asia and North America (Rydin and Jeglum 2006, Spitzer and Danks 2006). In terms of biogeography, peat bogs in Croatia represent a southern enclave of their northern continuous distribution (Topić and Stančić 2006). Peat bogs are highly endangered ecosystems in Europe (Holmes et al. 1993, Dapkus and Tamutis 2008, Buchholz et al. 2009) and some are listed as priority habitat types in Annex I of the European Habitats Directive (Anonymous 1992). Due to their small size, isolation, drainage and abandonment of traditional human activities, and especially to the progressive vegetation succession, such habitats are among critically endangered habitats in Croatia (Topić and Stančić 2006, Topić and Vukelić 2009).

These unique ecosystems often consist of specialized flora and fauna, largely limited only to these habitats. Although most of the peat matrix is dead, it contains a great variety of living organisms that contribute in decomposing (Rydin and Jeglum 2006). According to Peus (1932) and Roubal (1934) species that inhabit peat bogs can be classified into four categories: (a) tyrphobiontic species (occur only in bogs), (b) tyrphophilous species (characteristic of bogs but not strictly confined to them), (c) tyrphoneutral species (distributed across various types of habitats) and (d) tyrphoxenous species (vagrants or immigrants that cannot live in bogs).

As members of the soil macrofauna community, isopods play an important role in the processes and soil formation (Wolters and Ekschmitt 1997). As soil humidity is their essential requirement, they occur in microhabitats such as under stones and logs in leaf litter (Schmalfuss 1978). There are several papers about isopod fauna in Central European wetlands (Sallai 1993, Farkas 1998, Tajovský 1998, Vadkerti and Farkas 2002, Tuf 2003, Vilisics et al. 2007). However, isopod fauna on peatlands is basically unknown, especially in Europe.

The objectives of this study were: (1) to determine the terrestrial isopod assemblages and their spatial distribution in the peat bog remnant and surrounding forest, (2) to determine which basic ecological groups inhabit the peat bog and adjacent forest (3) to analyze the seasonal dynamics of the dominant isopod species and (4) to asses possible influence of soil temperature and humidity on isopod abundance.

## Methods

### Study area

Peat bog Dubravica is located in the Northwestern part of Croatia, in Hrvatsko zagorje Region (45°57.430'N, 14°44.470'E). It is situated in sessile oak and hornbeam forest (ass. *Epimedio-Carpinetum betuli* (Ht.1938) Borh. 1963) at an altitude of l60 m asl. Since 1966 it has been protected as a Botanical Reserve and it is a potential NATURA 2000 site.

According to Horvat (1939) there were three bogs that covered the area of 2566 m^2^ in total, but during the last 50 years the bog area has drastically decreased in size. There is only one small peat bog remnant covering the area of 605 m^2^ (Hršak 1996). This is mainly due to changes of the water level and abandonment of traditional management practices, such as mowing. All that led to the process of natural vegetation succession. A great part of the bog is overgrown by purple moor grass (*Molinia caerulea* (L.) Moench) and some woody species like the alder (*Alnus glutinosa* L.) and alder buckthorn (*Frangula alnus* Mill.). These changes in vegetation structure dry out and rise up the bog, enabling growth of less moisture substrate seeking plants (Hršak 1996). In order to preserve biodiversity of the peat bog remnant, the bog area is nowadays mowed once a year. Also, the presence of wild animals, like wild boar, brings to halting the spread of *Molinia* grass by rooting the land.

**Figure 1.**
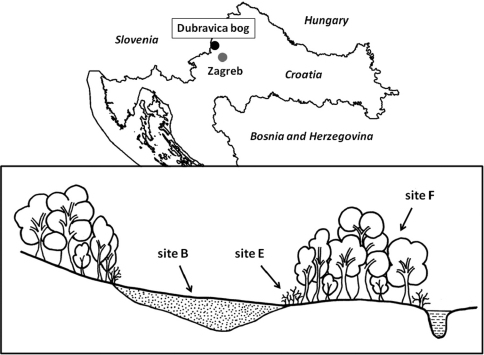
Sampling sites in the Dubravica bog and surrounding forest. **Site B** - bog **Site E** - edge **Site F** - forest.

Three sites were selected in and around the bog area, situated in different vegetational associations (Figure 1). The site B is located in the centre of the bog and the bog vegetation belongs to the *Rhynchosporetum albae* W. Koch, 1926 association. The ground layer was dominated by typical bog plant species: common sundew (*Drosera rotundifolia* L.), white beak-sedge (*Rhynchospora alba* (L.) Vahl.) and *Sphagnum* mosses (*Sphagnum subsecundum* Ness.). Site E is located on the edge of the bog and sessile oak and hornbeam forest. The edge is covered with rather strong *Rubus* sp. plants, which are opportunistically taking the open area on the edge zones. In order to avoid edge effect, site F is located 60 m from the edge into the forest (Lindenmayer and Fischer 2006). This site belongs to *Epimedio-Carpinetum betuli* association. Sessile oak (*Quercus robur* L.) and hornbeam (*Carpinus betulus* L.) are the dominant species in a tree layer.

### Sampling

Isopods were collected using pitfall traps during the carbide beetle study and sampling was conducted from May to November 2008 and from May to November 2009. Five plastic traps per site were placed (volume 0.3 dm^3^) 5 m apart. They were half filled with a saturated solution of sodium chloride with a few drops of detergent to break the surface tension of the liquid. A styrofoam roof was placed above each trap to protect them from rain. The traps were emptied once a month. The collected specimens were kept in 75% ethyl-alcohol with glycerol. The individuals were identified to the species level, apart from *Armadillidium* sp. and Oniscidae sp., due to lack of adult male individuals. All individuals were identified by authors (I. A. and J. B.), with the exception of *Hyloniscus adonis*, which was identified by Stefano Taiti (Istituto per lo Studio degli Ecosistemi, Consiglio Nazionale delle Ricerche).

### Soil analysis

Basic pedophysiological properties of the soil were determined using standard methods (Škorić 1982). Soil temperature and humidity were measured once a month at all three sites, next to every pitfall trap and average values were calculated. Temperature was measured at depth of 7 cm using P300 Dostmann electronic thermometer and soil humidity after every field trip by taking a soil sample at approximately 10 cm. In substrate samples water content was determined by the gravimetric method. The same soil sample was used to measure pH in water with a ratio of 1:2.5 (w/v) (10 g substrate / 25 mL H_2_O) and in KCl with a ratio of 1:2.5 (w/v) (10 g substrate / 25 mL KCl), using WTW pH 330i meter. Phosphorus, as P_2_O_5_ was measured by the colorimetric method using the spectrophotometer DR/2000 HACH (1996). Total nitrogen (TN) and total carbon (TC) content in substrate were simultaneously determined using the dry combustion method (elemental analysis) with the Vario Macro CHNS analyzer, Elementar.

**Figure 2.**
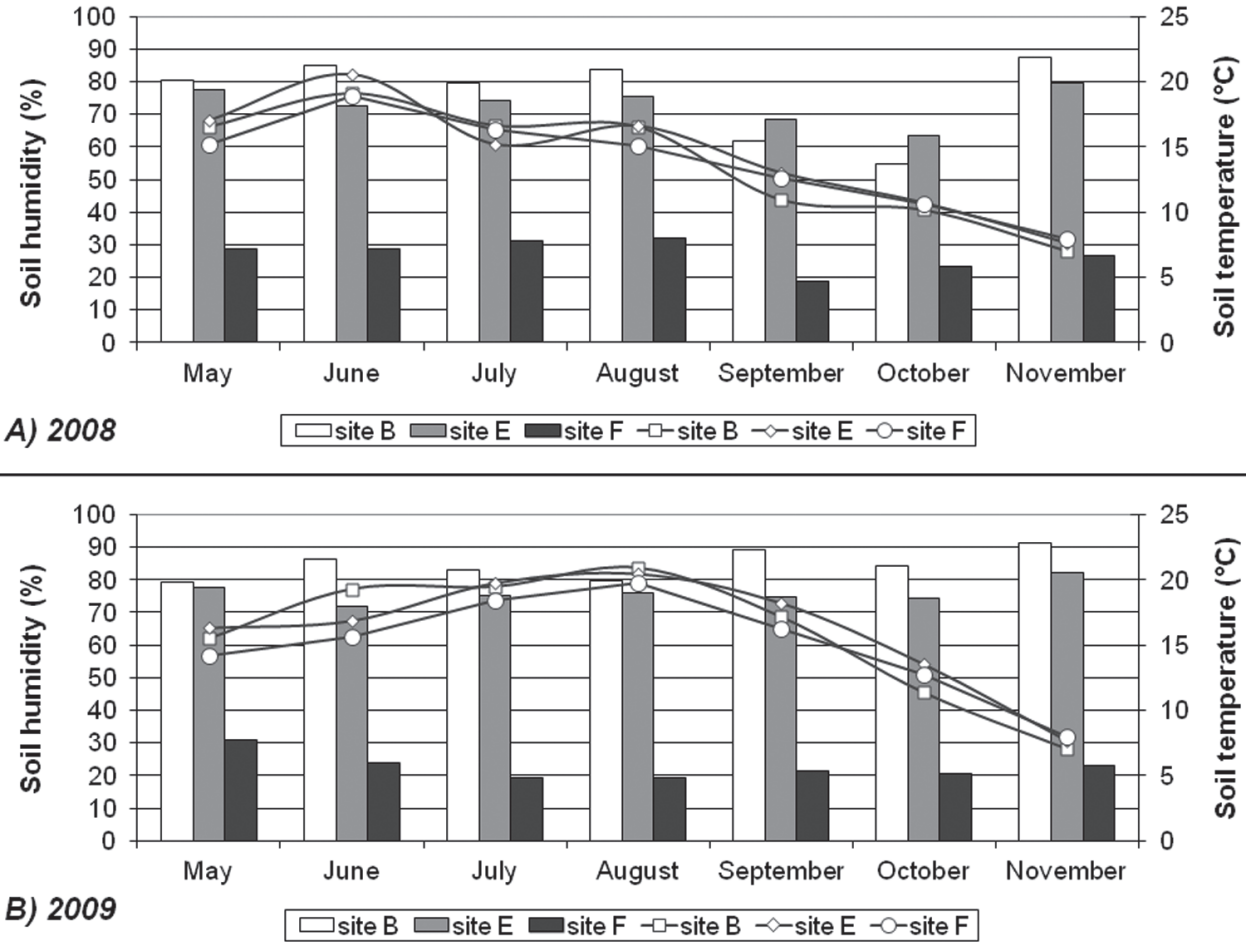
Soil temperature and soil humidity at the Dubravica bog and adjacent forest during 2008 and 2009. Temperature values are presented with different symbols and humidity values with bars. **Site B** – bog; **Site E** – edge; **Site F** – forest.

Soil analysis showed that pH values were very low at each sampling site, while calcium carbonate concentration was lowest in the forest, but higher at the edge and at the bog site (Table 1). Soil humidity was highest (sometimes ≥80%) at the bog during most of the sampling period. In addition, comparable results were observed at the edge, where soil humidity was also high (≥70%) (Figure 2). Soil humidity was particularly low in the forest (18–33%) in both study years. Oscillations of the soil temperature were most expressed at the edge (during both study years), especially during summer. At the bog an intermediate pattern was recorded (between edge and forest). Conversely, the lowest temperatures were measured at the bog site (during autumn and winter). Differences in soil humidity were observed in autumn between two study years, most likely due to higher amount of rain in 2009.

**Table 1.** Vegetational and pedagogical properties of studied sites in the Dubravica peat bog. **Site B** – bog; **Site E** – edge; **Site F** – forest.

**Environmental variable**	**Site B**	**Site E**	**Site F**
Vegetation analysis			
Plant association	*Rhynchosporetum albae* W. Koch	-	*Epimedio-Carpinetum betuli* (Ht 38) Both. 63
Vegetation height/m	1	0.2-0.5	20
Vegetation density	high	low	middle, thick layer of litter
Tree layer, dominant plant species	-	-	95% *Carpinus betulus*, *Quercus robur*
Frutescent layer, dominant plant species	0%	50% *Rubus* sp.	5% *Carpinus betulus*
Herbaceous layer, dominant plant species	100% *Sphagnum subsecundum*, *Molinia caerulea*	10% *Molinia caerulea*	80% *Epimedium alpinum*
Soil analysis			
Soil type	peat	-	stagnosol
pH (H_2_0)	4.47	4.18	4.09
pH (KCl)	3.92	3.55	3.57
Humus (Tjurin) %	7.0	6.3	2.0
P_2_O_5_ mg/100 g	0.8	1.7	0.4
CaCO_3_ %	0.235	0.226	0.127
C/N	17.2	15.6	13.6

### Data analysis

Activity-density was compared between two years, using Mann-Whiney U test and Spearman correlation. To calculate the diversity of the isopod assemblages we used Simpson (1-λ’) and Shannon-Wiener indices (H’). Evenness was estimated using Pielou’s evenness (J). Similarity between sites was calculated using the Bray-Curtis similarity coefficient, calculated on square root transformed and standardized abundance data (traps total catches). Non-metric MDS (multidimensional scaling) was constructed on Bray-Curtis similarity. The analyses were carried out using the PRIMER program v.6 (Clarke and Gorley 2006). Spearman’s rank correlation coefficient was used to identify relationship between isopod abundance and environmental parameters (soil temperature and humidity). Activity density data did not follow normal distribution (Shapiro-Wilk test, p<0.05), so square root transformation was applied (Shapiro-Wilk test, p>0.05). ANOVA was used to compare abundances of isopods (monthly data pooled for five traps) between sampling sites. Tukey HSD (Honestly Significant Difference) test was used for *post-hoc* comparisons. Normality of data was tested using Shapiro-Wilk W test. ANOVA was executed using Statistica 7.0 (StatSoft inc.). An independent and comparable habitat fidelity index was made for collected species for each habitat type. In the case of isopods from family Oniscidae sp. and *Armadillidium* sp. habitat fidelity index was not calculated due to small number of specimens caught. It was conducted according to Nyilas (1994), based on Erdakov et al. (1979). The formula was:

**Figure:**
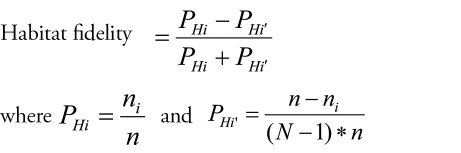


n_i_ = number of individuals in habitat; n: number of individuals in all habitats; N: number of habitats, Hi: habitat i, Hi’: all habitats except habitat i. The values of this index range from -1 to +1. The maximum value, +1 shows that all individuals of the given species were in the given habitat, whereas -1 indicates that no individuals of the species were recorded in the habitat, and 0 indicates that there was an average number of individuals in the habitat (Nyilas 1994). The sum of absolute values of the indices for a species gives the habitat selection index of the species.

## Results

### Species richness, activity density and diversity

During both study years, a total of 389 isopod individuals belonging to 8 species were captured (Table 2). Species richness and activity density were both lowest at the bog (over the whole study period). Overall, the highest number of individuals was recorded in the forest. ANOVA indicated significant difference in activity density between sites (d.f. = 2.42, F = 3.604, p<0.05). *Post-hoc* test separated activity density in the bog from values in the forest (Tukey HSD, p<0.05), while other combinations were not significantly different (Tukey HSD, p>0.05). Overall, activity density was positively correlated between the two years of the study. Moreover, no significant difference of the latter between 2008 and 2009 was recorded for site B (Mann-Whitney U-test, p=0.327), in contrast to sites E and F (Mann-Whitney U-test, p=0.013 and p=0.007). Spearman’s rank correlation coefficient was statistically significant for site B (R_s_=0.83, p=0.022), marginally significant for site E (R_s_=0.73, p=0.10) and not significant for site F (R_s_=0.55, p=0.20). Therefore, data for both years were pooled for subsequent analyses.

**Table 2.** Isopod species recorded at Dubravica bog and adjacent forest with indices of diversity and evenness. **Site B** – bog; **Site E** – edge; **Site F** – forest; **Tn** – tyrphoneutral species **Tx** – tyrphoxenous species; **%** – percent share of total individuals per site.

**Species name**	**site B**	**%**	**site E**	**%**	**site F**	**%**	**Ecological group**
*Armadillidium carniolense* Verhoeff, 1901	8	12.7	28	19.2	3	1.7	Tn
*Armadillidium* sp.	0	0.0	0	0.0	1	0.5	Tn
*Ligidium germanicum* Verhoeff, 1901	6	9.5	3	2.1	3	1.7	Tn
Oniscidae sp.	0	0.0	2	1.4	0	0	Tn
*Protracheoniscus politus* C. Koch, 1841	19	30.2	61	41.8	159	88.3	Tn
*Trachelipus rathkii* Brandt, 1883	28	44.4	20	13.7	3	1.7	Tn
*Trachelipus ratzeburgi* Brandt, 1883	1	1.6	17	11.6	8	4.4	Tn
*Hyloniscus adonis* Verhoeff, 1927	1	1.6	15	10.3	3	1.7	Tn (possible Tx)
Number of isopod species (S)	6		7		7		
Number of isopod individuals (N)	63		146		180		
Simpson index (1-λ’)	0.697		0.750		0.218		
Shannon-Wiener index (H’)	1.339		1.576		0.549		
Pielou’s evenness index (J’)	0.748		0.810		0.283		

Soil temperature was positively correlated with the isopod total monthly catch at all three studied sites (Spearman’s rank correlation; site B: N=14, R_s_=0.594, p=0.025; site E: N=13, R_s_=0.619, p=0.024; site F: N=14, R_s_= 0.558, p=0.038). On the contrary, soil humidity negatively correlated with the isopod total monthly catch in the bog and forest samples, but the correlations were not statistically significant (Spearman’s rank correlation; site B: N=14, R_s_= -0.246, p=0.397; site F: N=14, R_s_= -0.372, p=0.190). However, in edge samples correlations between soil humidity and the total monthly catch was positive, but also statistically not significant (Spearman’s rank correlation; site E: N=13, R_s_=0.077, p=0.802).

*Protracheoniscus politus* C. Koch was generally the most abundant species (61.4% of the total catch). Together with following species; *Trachelipus rathkii* Brandt (13.1%), *Armadillidium carniolense* Verhoeff (10%) and *Trachelipus ratzeburgi* Brandt (6.7%) it constituted 91.2% of the total catch. *Trachelipus rathkii* was the most abundant species at the bog (44.4% of the catch), while its abundance decreased at the edge, and was the lowest in the forest (reaching only 1.7%). Three following species; *Protracheoniscus politus* (30.2%), *Armadillidium carniolense* (12.7%) and *Ligidium germanicum* Verhoeff (9.5%) also accounted for a larger proportion of the catch at the bog. *Protracheoniscus politus* (41.8%) and *Armadillidium carniolense* (19.2%) were the most frequent species at the edge and *Protracheoniscus politus* was particularly abundant in the forest (88.3%). *Hyloniscus adonis* Verhoeff,had the highest abundance at the edge, while only 1 specimen was found at the bog. Majority of isopod taxa found at the bog are tyrphoneutral. According to diversity and evenness indices, the greatest and lowest diversity was recorded at the edge and in the forest, respectively.

### Seasonal activity

**Figure 3.**
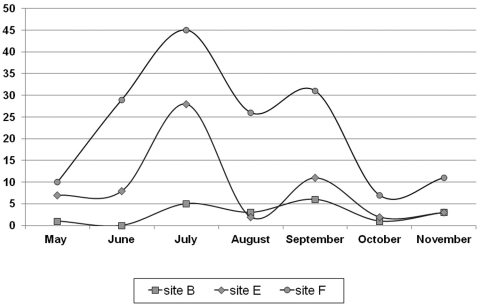
*Protracheoniscus politus* seasonal activity at three studied sites with different vegetation. **Site B** – bog; **Site E** – edge; **Site F** – forest; Y-axis shows total number of monthly caught individuals.

Activity density of dominant *Protracheoniscus politus* was expressed as the total number of individuals caught monthly and plotted against time (Figure 3). The maximum seasonal activity was observed in July at both forest and edge sites. During August, the number of individuals decreased at all studied sites, whereas an increase was observed in September. Overall, low number of individuals was caught at the bog, and this was insufficient to observe seasonal dynamics. Seasonal activity density of *Protracheoniscus politus* did positively correlate with soil temperature at the edge and forest sites (Spearman’s rank correlation; site E: N=14, R_s_=0.093, p=0.751; site F: N=14, R_s_=0.427, p=0.128), and negatively at the bog (Spearman’s rank correlation; site B: N=14, R_s_= -0.168, p=0.565). However, the correlations were not statistically significant. Additionally, Spearman’s rank correlation coefficient was positive between soil humidity and seasonal activity density of *Protracheoniscus politus* at the bog and edge (site B: N=14, R_s_=0.025, p=0.932; site E: N=14, R_s_=0.033, p=0.910, respectively) and negative in the forest (site F: N=14, R_s_= -0.461, p=0.096). Also, correlations were not statistically significant.

### Similarity and habitat fidelity

The habitat fidelity values of collected isopod species are shown in Table 3. According to these results, two dominant species, *Protracheoniscus politus* and *Trachelipus rathkii*, preferred different habitat types. *Protracheoniscus politus* mostly inhabited forest (site F) with a high habitat fidelity value (+ 0.6), while positive habitat fidelity value was calculated for *Trachelipus rathkii* (+ 0.4) at the bog site. Typical hygrophylic species, *Ligidium germanicum* and *Hyloniscus adonis* also showed quite different habitat preferences. *Ligidium germanicum* occurred mostly at the bog area (+ 0.33), while *Hyloniscus adonis* preferred the edge site (+ 0.77). *Armadillidium carniolense* and *Trachelipus ratzeburgi* seemed both to prefer edge site with a higher habitat fidelity values in contrast to the values calculated for the other sites.

**Table 3.** The habitat fidelity values of dominant isopod species in each of the three habitats. **Site B** – bog; **Site E** – edge; **Site F** – forest.

	**∑ N**	**site B**	**site E**	**site F**	**Habitat selection index**
*Protracheoniscus politus*	239	- 0.7	- 0.05	+ 0.6	1.35
*Trachelipus rathkii*	51	+ 0.4	+ 0.1	- 0.8	1.3
*Armadillidium carniolense*	39	- 0.32	+ 0.67	- 0.73	1.72
*Trachelipus ratzeburgi*	26	- 0.88	+ 0.54	- 0.06	1.48
*Hyloniscus adonis*	19	- 0.8	+ 0.77	- 0.47	2.04
*Ligidium germanicum*	12	+ 0.33	- 0.2	- 0.2	0.73

**Figure 4.**
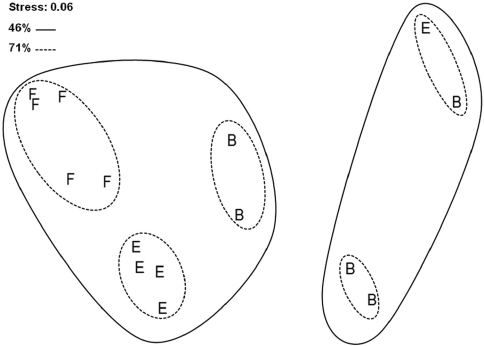
nMDS ordination of studied sites and Bray-Curtis similarities with superimposed results of cluster analysis. **Site B** – bog; **Site E** – edge; **Site F** – forest.

Non-metric MDS ordination based on Bray-Curtis similarity index, with superimposed results of cluster analysis (group-average linking), shows generally high degree of similarity between studied sites (i.e. they all cluster at 46% similarity level; Figure 4). In particular, high degree of similarity is obvious for forest and edge sites that cluster together at 71% similarity level. Forest replicate samples grouped strongly together at the same similarity level, whereas, most of the edge replicate samples clustered together at 80% similarity level. On the contrary, bog replicate samples did not form a distinct group and they were combined with edge samples.

## Discussion

Current study shows that isopod species richness of the isolated bog was surprisingly high and it did not considerably differ from the species richness of the edge or adjacent forest. Contrary to this, Peus (1932) found that isopods may be almost completely excluded from acidic bogs, mainly due to specific and rather extreme environmental conditions, particularly low acidity. The same was observed in isopod studies in the Canadian peat bogs (Judd 1963, Blades and Marshall 1994). According to Judd (1963) only two isopod species were recorded at the Byron bog, but none of them was found in the open bog area, than on the adjacent wooded slopes. However, our study shows completely different pattern. This is possibly a result of several factors including progressive vegetation succession, low water table and small size of the bog, thus enabling immigration from adjacent habitats. In contrast to northern acidic peat bogs where water level is at or near the surface of the substratum and the peat is infra aquatic, water level at the studied bog has considerably fallen under the minimum necessary level to prevent spreading of taller plants, such as purple moor grass (Hršak 1996). Consequently, *Sphagnum* hummocks became less humid and such changes might have in particular affected isopod presence at the bog. Another cause might be the latitude, since generally isopod species richness increases from north to south (Hornung and Sólysmos 2007), and the same pattern was observed for e.g. spiders (Koponen et al. 2001, Koponen 2002) and ants (Gotelli and Ellison 2002) in peat bogs. Comparing our results with recent study of Farkas and Krčmar (2004) in the Danube and Drava floodplains where 11 isopod species were recorded using more pitfall traps and covering more different habitats, it seems that isopod diversity of the isolated Dubravica bog is not negligible.

The greatest diversity of isopods in the current study was found exactly in the edge mainly due to the edge effect. According to Hilty et al. (2006) ecotones are sites of high productivity and enhanced biodiversity. Furthermore, species richness of carabid beetles and ants were also highest at the edge in the Dubravica bog (Brigić et al. 2009, Bujan et al. 2010). According to the nMDS analysis, forest and edge sites show high degree of similarity most likely due their vicinity. Although these two sites differ considerably in soil humidity, it seems that forest isopod species can successfully disperse into or even inhabit the edge zone. Bog replicate samples do not form a firm group and cluster near edge sites most likely due to the process of immigration.

In our study, isopod activity density significantly differed between the bog and surrounding habitat (forest), being the lowest at the bog. This is in accordance with data from studies on other ground dwelling arthropods in peat bogs, e.g. carabid beetles (Främbs et al. 2002, Nietupski et al. 2008). Most likely, the findings are influenced by sampling method, environmental variables (such as soil humidity, pH values, vegetation structure and density) and predation pressure. Pitfall traps are most commonly used in various ecological studies of ground dwelling arthropods, such as carabid beetles (Thiele 1977, Spence and Niemelä 1994) or spiders (Uetz and Unzicker 1976). However, pitfall trapping depends on the activity of an organism (Thiele 1977), therefore factors influencing arthropod activity, also affect the number of caught individuals. Consequently, cryptozoic isopod species that live under the stones, tree bark or in the soil were under sampled or are missing from our list (e.g. (*Trichoniscus* spp., *Haplophthalmus* spp.). Furthermore, lower isopod activity might be caused by the surface structure of *Sphagnum* mosses creating a thick carpet and hence some isopod species may not be recorded. Furthermore, heavy and large bodied bumbling isopods would easily fall into the pitfall traps, whereas smaller ones would more likely be able to stop themselves on the edge (Sutton 1972).

The harsh environmental conditions, such as daily and annual temperature differences and low pH values (Figure 2; Table 1) might also have affected the isopod species richness and activity at the bog (Zimmer et al. 2000, Tuf and Jeřábková 2008). Mostly due to large calcium demands for cuticular calcification (Sutton 1972), isopods prefer to inhabit more alkaline than acid habitats (Wolters and Ekschmitt 1997). Hygropositive and photo-negative behaviour is known in isopods (Gunn 1937, Waloff 1941) enabling them to inhabit most favourable environments. Our study reveals that isopod activity (total monthly catch) was positively correlated with soil temperature. Soil temperature fluctuates annually and daily and it is affected mainly by variations in air temperature (Zehng et al. 1993). Positive correlation could be the result of higher isopod’s activity during colonisation of more suitable microhabitats (i.e. from hot and drier environments to the ones with higher humidity level and lower temperatures; Edney 1953). Similar trend was observed in correlation of isopod activity with soil humidity. Gunn (1937) and Waloff (1941) have both shown that isopod’s activity has decreased at high humidity conditions. These conditions were present at the bog area, where we collected low number of isopod individuals. However, in the forest, pitfall traps were probably positioned in microhabitats with optimum ecological conditions and therefore higher numbers of individuals were collected. A comprehensive study applying additional sampling methods, such as hand collecting or extracting from soil samples with Tullgren apparatus, should provide more precise results.

Except above mentioned grounds, predation might also influence isopod activity density at the bog. High population density of lycosid spiders (Štambuk and Erben 2002) and ants (Bujan et al. 2010) were found at the studied bog. Both arthropod groups are common predators on isopods (Sutton 1970, Sunderland and Sutton 1980, Deslippe et al. 1996). According to Bujan et al. (2010) extremely high number of the ants was caught at the studied bog, precisely 13 584 individuals (while at both edge and forest sites less than 1000 individuals). Two ant species, *Myrmica rubra* L. and *Myrmica ruginodis* Nyl., accounted for 99% of the catch. These species prefer wet habitats (Seifert 1988) and their great colony density is most likely caused by specific way of colony founding-colony budding (Steiner et al. 2005), wherefore there is one big anthill present at the bog (Bujan et al. 2010). Castillo and Kight (2005) showed that ant presence can influence both isopod behaviour (defensive and aversive behaviour) and reproductive success (shortening brooding period). Concerning the spiders, lycosid spiders were dominant members of the spider fauna at the bog and they made 72.7 % of the total catch (Štambuk and Erben 2002). According to Sutton (1970) they have a low food capacity for isopods, although in a combination of their high population density and small living area they could be considered as significant predators on isopods.

*Trachelipus rathkii* was the dominant species at the bog. Its abundance decreased towards the forest where the soil humidity was lower comparing to other two sites. Furthermore, its habitat fidelity value was highest at the bog (Table 3). As a widely distributed and common isopod species, *Trachelipus rathkii* inhabits different biotopes, including extreme ones (Gruner 1966), but prefers open places, and it is usually not found within woods (Schmidt 1997), which is in accordance with our study. This species is typical for flooded ecosystems (Farkas 1998, Tuf and Tufová 2005) and it is common inhabitant of wetlands and peat bogs (Potočnik 1993). Throughout the experiments, it was observed that *Trachelipus rathkii* has a high ability to survive under water and can therefore colonise such habitats (Tufová and Tuf 2005). It was also found in few other bogs in Croatia (Antonović, unpublished data). However, Judd (1963) recorded this species on the wooded slopes with no records in the bog area. However, that bog area was completely flooded and unfavourable for any terrestrial isopod species. In contrast to *Trachelipus rathkii*, *Trachelipus ratzeburgi* is a sylvan species (Farkas et al. 1999, Tuf and Tufová 2005, Farkas and Vilisics 2008), found in relatively low numbers in all explored sites, with highest abundance at the edge.

Although species from the family Trichoniscidae, including *Hyloniscus* spp. are highly hygrophylic, and usually inhabit wetlands, species *Hyloniscus adonis* is not abundant on the bog, but surprisingly on the edge. Due to bog’s inclination towards the edge, high, but relatively even soil humidity was recorded throughout the sampling period at the edge site. Hence, availability of microhabitats with optimum ecological conditions for this species, but also lower predation pressure from ants and lycosid spiders could explain its highest abundance at the edge.

Among typical central European species, rather rare and less known isopod *Ligidium germanicum* was present at all study sites. It is highly hygrophylic species (Potočnik 1993), therefore the highest abundance at the bog site is expected.

At all studied sites, *Protracheoniscus politus* was one of the most frequent and numerous species. It was dominant species in the forest where the soil humidity content was considerably lower. It is a xerophilous species that prefers dryer habitats (Potočnik 1993). Additionally, it is sylvan species (Karaman 1974, Tomescu et al. 2008) inhabiting woodlands, where it is a constant and dominant element (Karaman 1974, Farkas and Krčmar 2004, Tuf and Tufová 2005, Farkas and Vilisics 2008). Therefore, it is not surprising that its habitat fidelity value was highest in the forest (Table 3). *Protracheoniscus politus* was also found in the bog. It seems that this species can easily enter the bog area especially during the summer, most likely due to the size of the bog and progressive vegetation succession (Figure 3). The activity of *Protracheoniscus politus* was the highest in July and September. The soil temperatures in summer were the highest, but Spearman’s rank correlation coefficient was not statistically significant, therefore soil temperature is not the key factor for activity differences. The latter is in accordance with results of Oberfrank et al. (2011). The population study from north western Romania showed also two peaks in population’s dynamics, the first in April-May-June and the second in September-October (Radu and Tomescu 1972). Such seasonal activity could possibly be explained by the facultative iteroparous univoltine reproductive strategy of *Protracheoniscus politus*, having the reproductive season from May to August. Temporal pattern of sex ratio has its maximum (0.98 for males) by the end of April, during males-search and copulation, but the overall yearly sex ratio was around 0.27. Similar as in our study, the second reproductive peak with postmarsupial females was in July, and total female activity was highest in September (Oberfrank et al. 2011). The presence of *Armadillidium carniolense*, a further sylvan species (Tomescu et al. 2002), at the bog area is an additional evidence of strong impact of vegetation succession on the bog, changing it into a drier habitat. Similar was observed by Štambuk and Erben (2002) during the study of the lycosid fauna. Although the lycosid fauna of the bog was relatively bog-specific, changes in the habitat structure resulted in higher abundance of some forest species (Štambuk and Erben 2002).

Within the current and previous studies (e.g. Judd 1963, Blades and Marshall 1994), typical tyrphobiontic and tyrphophilous species were not recorded. On the contrary, *Hyloniscus adonis* could be considered as a potential tyrphoxenous species, since it showed some habitat preferences. Isopod fauna of peatlands is generally poorly investigated, hence further comprehensive studies providing more data on ecology of isopods inhabiting peat bogs are necessary.

## Conclusions

This study reveals that vegetation succession has a strong impact on community composition of fauna inhabiting the peat bog. With overgrowth of the peat bog by *Myrmica caerulea* grass, water level has significantly decreased. Therefore, the bog area becomes dryer, shaded and more suitable for forest species. Although there were no previous studies on isopod fauna, the presence of forest species indicates such changes in this habitat. Peat bog size and interspecific relationships, such as predation, also affected isopod species richness, activity density and diversity. Typical tyrphobiontic and tyrphophilous species were not observed, but further studies implying additional sampling methods should provide more detailed insight into isopod faunistics and ecology. In order to preserve suitable microclimatic conditions and biodiversity of the bog, management practices, like mowing, are required.
